# A comparison of two methods for estimating prevalence ratios

**DOI:** 10.1186/1471-2288-8-9

**Published:** 2008-02-28

**Authors:** Martin R Petersen, James A Deddens

**Affiliations:** 1 Division of Surveillance, Hazard Evaluations, and Field Studies, National Institute for Occupational Safety and Health, Mail Stop R15 4676 Columbia Parkway Cincinnati, OH 45226, USA; 2 Department of Mathematical Sciences, University of Cincinnati, Cincinnati, Ohio, USA

## Abstract

**Background:**

It is usually preferable to model and estimate prevalence ratios instead of odds ratios in cross-sectional studies when diseases or injuries are not rare. Problems with existing methods of modeling prevalence ratios include lack of convergence, overestimated standard errors, and extrapolation of simple univariate formulas to multivariable models. We compare two of the newer methods using simulated data and real data from SAS online examples.

**Methods:**

The Robust Poisson method, which uses the Poisson distribution and a sandwich variance estimator, is compared to the log-binomial method, which uses the binomial distribution to obtain maximum likelihood estimates, using computer simulations and real data.

**Results:**

For very high prevalences and moderate sample size, the Robust Poisson method yields less biased estimates of the prevalence ratios than the log-binomial method. However, for moderate prevalences and moderate sample size, the log-binomial method yields slightly less biased estimates than the Robust Poisson method. In nearly all cases, the log-binomial method yielded slightly higher power and smaller standard errors than the Robust Poisson method.

**Conclusion:**

Although the Robust Poisson often gives reasonable estimates of the prevalence ratio and is very easy to use, the log-binomial method results in less bias in most common situations, and because it fits the correct model and obtains maximum likelihood estimates, it generally results in slightly higher power, smaller standard errors, and, unlike the Robust Poisson, it always yields estimated prevalences between zero and one.

## Background

The most common method of modeling binomial health data in cross-sectional studies today is logistic analysis. It was first used to replace probit analysis for bioassay data sixty years ago by Joseph Berkson [1]. Weighted least squares, minimum logit, and maximum likelihood methods were developed and used by various investigators to estimate the parameters [1-10]. Maximum likelihood was theoretically the best estimation method, and as computer programs were written to obtain its iterative solutions, maximum likelihood became the method of choice.

Logistic analysis works very well if one wants to model the ratio of odds instead of the ratio of probabilities. It also yields a good approximate analysis if one is interested in the ratio of probabilities of a rare disease. However, if the disease is not rare, and one is interested in the ratio of probabilities, then the logistic approximation will be poor because the odds ratio will be a poor estimator of the probability ratio. For example, if 80 out of 100 exposed subjects have a particular disease and 50 out of 100 non-exposed subjects have the disease, the odds ratio (OR) is 4, but the exposed subjects are only 1.6 times as likely to have the disease as the non-exposed subjects. Thus, any author or reader, who considers exposure to be related to a four-fold increase in the chances of getting the disease, would be substantially overestimating the effect of the exposure. The number 1.6 (for this example) can be called the probability ratio, the proportion ratio, or in studies of existing disease, the prevalence ratio (PR). The latter will be used in this paper.

In this example, the larger of the two prevalences is 0.80. If the prevalence ratio is 1.6, then in order for the odds ratio to be within 10% of the prevalence ratio (i.e. for the odds ratio to be no more than 1.76), the larger of the two prevalences can be no more than 0.2105. This number decreases as the prevalence ratio increases. Thus it is difficult to define "rare" in general. However, for prevalence ratios up to 10, if both prevalences are no larger than 0.10, then the odds ratio will be within 10% of the prevalence ratio. For prevalences larger than 0.10, it is safer to estimate the prevalence ratio directly.

Logistic analysis has been a popular analysis tool for cross-sectional studies because 1) standard statistical software packages perform logistic analysis; 2) if the disease or outcome is rare, then odds ratios are approximately equal to prevalence ratios; and 3) if the disease is not rare, then there have not been any good alternatives. The latter has changed, however, because most standard statistical software packages now perform generalized linear modeling, which includes, among other things, linear, logistic, Poisson, and log-binomial modeling.

Skov et al. recommended using the log-binomial model, which directly models the prevalence ratio [11]. If for each combination of independent variables, the dependent variable has a binomial distribution with the logarithm of its probability being linearly related to the independent variables, then the log-binomial is the correct model, and maximum likelihood estimates of the parameters and prevalence ratio can be directly obtained. However, for many situations with quantitative covariates, the maximum likelihood estimate (MLE) is on the boundary of the parameter space. For many software packages, the model fails to converge because the instantaneous slope of the likelihood may not be zero on this boundary. Thus standard software packages which maximize the likelihood by finding the point at which the derivative is equal to zero may not work properly.

Deddens et al. extended Skov's maximum likelihood solution to situations in which the MLE is on the boundary of the parameter space [12]. The method, called the COPY method, gives very good approximate MLEs. It involves using the MLEs when the log-binomial model converges, and, when it does not converge, using MLEs from a new data set that contains c-1 copies of the original data and 1 copy of the original data with the dependent variable values interchanged (1's changed to 0's and 0's changed to 1's). For any finite c, the solution is no longer on the boundary, and thus the solution is an MLE for this data set. As c gets large, the MLE estimates for this modified data set approach the MLEs for the original data set. The number c should probably be at least 100. In this paper, c = 1,000 was always used.

Lee and others recommended using the Cox proportional hazard model to estimate the prevalence ratio [13,14]. This method yields partial likelihood estimates of linear model coefficients except for the intercept which is not estimated. Skov et al., Deddens et al., and Barros and Hirakata showed that the Cox method yields estimated standard deviations which are too large, which leads to low power for Wald tests [11,12,15].

It is well known that when the prevalence is low and the sample size is large, probabilities from the Poisson distribution can often be used to approximate probabilities from the binomial distribution. Similarly, one can think of an existing sample of binomial data (0 or 1) as being approximately Poisson, where the probability of a value of 2 or greater is low enough that no values greater than 1 occurred in the obtained sample. By assuming that the logarithm of the Poisson parameter (mean) is linearly related to a set of independent variables, the exponentiation of any coefficient of the model will yield an estimate of a ratio of Poisson parameters. Because the observed data consist of only zeros and ones, this ratio can be used as an approximation to the prevalence ratio. Assuming equal follow-up times for all subjects and handling ties properly, the partial likelihood estimates and estimated standard errors of the non-intercept parameters from Cox proportional hazard regression are exactly the same as the estimates from Poisson regression [16]. Thus, Poisson regression suffers from the same problem (large standard errors) as the Cox model. For the most part, Poisson regression will be discussed in this paper rather than Cox proportional hazard regression because the intercept is estimated.

Barros and Hirakata have suggested methods involving robust variance estimation which appear to solve the large variance problem for Poisson regression [15]. They compared methods of adjusting the scale parameter in Poisson regression, but concluded that the best adjustment was to use a sandwich estimator of the variance. This "Robust Poisson" method represents a vast improvement over the regular Poisson method. In an independent investigation, Zou later suggested using this sandwich estimator and showed how to use PROC GENMOD in SAS to obtain it [17].

Another method to estimate the prevalence ratio is the direct conversion of an odds ratio to a prevalence ratio, which McNutt et al. showed is fairly biased when adjusted for other covariates [18,19]. Thompson et al. also discussed direct estimation of the PR from the OR, as well as weighted averages of these estimated PR's over strata and the stratified Mantel-Haenszel estimate [20]. Of the methods available at that time, they recommended using either the proportional hazards (i.e. Poisson) or the log-binomial method.

Schouten et al. suggested modifying the data in such a way that the odds ratio from logistic analysis for the modified data is an estimate of the prevalence ratio for the original data [21]. This estimator combined with a robust variance estimator yields a method which is similar to the Robust Poisson method, in that it uses a robust variance estimator, and to the COPY method, in that it uses data manipulation to obtain the result. Skov et al. showed that this estimator generally gave good results, but that sometimes the estimated probabilities could be greater than one [11]. Thus Skov recommended the log-binomial method.

There is much misinformation in the literature concerning which methods can yield probability estimates outside the range of zero to one. By definition, maximum likelihood estimates for binomial models cannot yield estimates of probabilities outside this range (because the probability estimates are MLEs also). Thus Skov's method for fitting the log-binomial model cannot yield such estimates. Similarly, the COPY method cannot yield such estimates, because it uses Skov's method on a data set modified so that the MLE is inside the parameter space. It is known, and will be shown again in this paper, that the Poisson and Robust Poisson can yield such invalid probability estimates [22,23].

Both the log-binomial method and the Poisson method are generalized linear models with a log link function, which is assumed to be the correct form. For each combination of independent variables, the distribution of the dependent variable is assumed to be binomial. The Poisson model erroneously treats this distribution as Poisson, and the log-binomial correctly treats it as binomial. In this paper, we compare these two methods: (1) the maximum likelihood estimates and likelihood ratio tests for the log-binomial model, using the COPY method to solve any convergence problems, with (2) the Poisson based estimators and Wald tests, using a sandwich estimator to solve the large variance problem. Although it is clear that the log-binomial and COPY methods should yield better estimates than the Poisson methods, we will use some limited simulations to illustrate the amount of this superiority and also indicate some situations in which the Poisson methods might be preferred. In addition, we will illustrate the use of both methods on real data sets.

## Methods

Comparisons between the Robust Poisson and log-binomial methods were made using simulated and real data sets. The simulations were a repeat of some of those performed by Deddens et al. Specifically, they were performed for the situation of one continuous covariate, *X*, with *X *uniformly distributed from 0 to 10 [12]. At each value of *X*, a value of *Y *was randomly generated from a binomial distribution with a sample size of 1 and a prevalence of exp(β_0 _+ β_1_*X*). The prevalence at *X *= 5 varied among 0.3, 0.5, and 0.7. Three values were chosen for β_1_, namely zero, medium, and large, where medium and large depended on the prevalence. The intercept, β_0_, was then determined from the prevalence at *X *= 5 and the slope, β_1_. Thus, there were nine basic simulations, and the sample size was set at n = 100 for each simulation. This sample size was chosen because it was felt to be large enough for large sample properties to hold, but not so large that both methods would have power too high for comparison. The data (same *X*'s, different *Y*'s) were replicated 1,000 times for each simulation. Although logistic analysis is reasonable when the prevalence is 0.1, and a prevalence of 0.9 is unusual, simulations with n = 100 were also performed for these prevalences. Simulations were repeated for these two prevalences with a zero and a large slope for n = 1,000. For all simulations, in addition to estimates from the log-binomial and Robust Poisson methods, exact maximum likelihood estimates were obtained (even when convergence was not obtained on the original data) [12]. This was accomplished using the macro supplied by Deddens et al. [12]. Briefly the macro finds the point on the boundary, and it restricts the search for the MLE to parameters which force the likelihood through this point. All hypothesis tests were considered significant if the p-value was less than or equal to 0.05. Likelihood ratio tests were performed for the log-binomial method. When the data were copied 1,000 times, it was necessary to multiply the standard errors by the square root of 1,000 and to divide the log likelihoods by 1,000 for the likelihood ratio tests [12]. Wald tests were used for the Poisson method.

The real data sets come from on-line SAS examples [24]. Example 1, from the SAS PROC LOGISTIC documentation, is a study of the effects of rate and volume of air on a transient reflex vaso-constriction of the digit skin. A binomial variable for vaso-constriction (constricted = 1, not constricted = 0) was modeled on the logarithm of air rate and the logarithm of air volume using 39 trials. Although this was used by SAS to illustrate logistic analysis, the prevalence of vaso-constriction was .51, so odds ratios would not be good estimates of prevalence ratios.

Example 2, also from the SAS PROC LOGISTIC documentation, is a study of the analgesic effects of treatments on 60 elderly patients with neuralgia, in which a binomial variable for pain (no pain = 1, pain = 0) is modeled on treatment (3 levels), gender (2 levels), and age (years) [24]. Although this was used by SAS to illustrate logistic analysis, the prevalence of no pain was .58, so odds ratios would not be good estimates of prevalence ratios.

Example 3 comes from a book by Paul Allison, but it is also available online [25,26]. These data come from a study relating death penalty (death = 1, life in prison = 0) to defendant race (2 levels), victim race (2 levels), crime seriousness (quantitative scale), and culpability (quantitative scale). Although this was used by Allison to illustrate logistic analysis, the prevalence of death was .34, so odds ratios would not be good estimates of prevalence ratios.

All analyses were performed using SAS [24,27]. The Robust Poisson method was performed with PROC GENMOD using the REPEATED option [17]. The log-binomial method was performed with the macro from Deddens et al., which in turn used PROC GENMOD (with 1,000 copies when the model failed to converge) [12].

## Results

The estimates obtained using the log-binomial and Robust Poisson methods for the simulated data are shown in Table 1. The size (the probability of concluding that the true slope is not zero when in fact it is zero) and power (the probability of concluding that the true slope is not zero when in fact it is not zero) estimates for the tests in the simulations are shown in Table 2. In the simulations, the log-binomial method, using the COPY method approximations as needed, gave results which were very close to the exact maximum likelihood estimates, size, and power. Thus the exact results are not included in Table 1 and Table 2. The parameter of most interest is the slope, which is the logarithm of the prevalence ratio. The log-binomial and Robust Poisson estimates are close to the true parameters (Table 1). For prevalences of 0.3 and 0.5, the log-binomial method appears to be less biased for estimating the slope, while for a prevalence of 0.7, the two methods were about equally biased. However, these differences aren't apparent until the third, or more often, the fourth decimal place, so both methods work well. When the prevalence was 0.1, the log-binomial method had less biased estimates (not shown) and when the prevalence was 0.9, the Robust Poisson method generally had less biased estimates (not shown). The log-binomial method usually yielded slightly smaller estimated standard errors.

**Table 1:** Average log-binomial method and Robust Poisson method estimates*

		Zero Slope		Medium Slope		High Slope	
Prevalence at *X *= 5		Intercept (SE)^†^	Slope (SE)	Intercept (SE)	Slope (SE)	Intercept (SE)	Slope (SE)

0.3	True Parameters	-1.2040	0.00	-1.7040	0.10	-2.2040	0.20
		(Conv. = 100%)^‡^		(Conv. = 99.9%)		(Conv. = 90.9%)	
	Log-Binomial	-1.2292 (0.3250)	0.0001 (0.0559)	-1.7387 (0.3692)	0.1016 (0.0542)	-2.2512 (0.3900)	0.2046 (0.0488)
	Robust Poisson	-1.2291 (0.3247)	0.0001 (0.0558)	-1.7426 (0.3692)	0.1023 (0.0544)	-2.2634 (0.4027)	0.2064 (0.0520)

0.5	True Parameters	-0.6931	0.00	-0.9431	0.05	-1.1931	0.10
		(Conv. = 100%)		(Conv. = 99.8%)		(Conv. = 93.5%)	
	Log-Binomial	-0.7086 (0.2109)	0.0014 (0.0361)	-0.9512 (0.2297)	0.0501 (0.0352)	-1.2039 (0.2413)	0.1006 (0.0327)
	Robust Poisson	-0.7088 (0.2112)	0.0015 (0.0362)	-0.9517 (0.2311)	0.0502 (0.0356)	-1.2058 (0.2477)	0.1009 (0.0345)

0.7	True Parameters	-0.3567	0.00	-0.5067	0.03	-0.6567	0.06
		(Conv. = 99.0%)		(Conv. = 96.1%)		(Conv. = 70.3%)	
	Log-Binomial	-.3686 (0.1374)	0.0010 (0.0236)	-0.5115 (0.1485)	0.0297 (0.0226)	-0.6579 (0.1509)	0.0598 (0.0194)
	Robust Poisson	-.3680 (0.1383)	0.0009 (0.0237)	-0.5139 (0.1513)	0.0301 (0.0234)	-0.6669 (0.1621)	0.0614 (0.0225)

* Based on 1,000 simulations of the log-binomial model with a sample size of 100 and a single independent variable, *X*, with uniform distribution [0, 10]. The log-binomial method used the COPY method approximation when needed.

^† ^Standard Error.

^‡ ^Percentage of times the log-binomial model converged on the original data.

**Table 2:** Estimated size and estimated power for log-binomial and Robust Poisson methods*

Prevalence at *X *= 5	Method	Zero Slope	Medium Slope	High Slope
		
		Size^†^	Power^†^	Power^†^
0.3	Log-Binomial	0.054	0.477	0.989
	Robust Poisson	0.051	0.461	0.984
0.5	Log-Binomial	0.049	0.279	0.856
	Robust Poisson	0.050	0.275	0.842
0.7	Log-Binomial	0.045	0.256	0.825
	Robust Poisson	0.045	0.258	0.815

* Same simulations as in Table 1. Estimated size and power are the proportions of the 1,000 simulations which have a p-value less than or equal to 0.05. The log-binomial method used the COPY method approximation when needed. Wald tests were used for the Robust Poisson method, and likelihood ratio tests were used for the log-binomial method.

^† ^Size is the probability of concluding that the true slope is not zero when in fact it is zero, and power is the probability of concluding that the true slope is not zero when in fact it is not zero.

The estimated sizes for both methods were approximately correct (Table 2). The estimated powers were generally higher for the log-binomial method, but only slightly so. The pattern for the estimated powers was the same when the prevalence was 0.1 or 0.9 (not shown). However, when the prevalence was 0.1, the estimated sizes were 0.061 for the log-binomial method and 0.069 for the Robust Poisson method, which were slightly too high.

When n = 1,000 (not shown), the estimates for the log-binomial and Robust Poisson methods were essentially the same. When the slope was large, the Robust Poisson had a slightly larger estimated standard error for the slope. All of the sizes were close to 0.05, and all of the powers were 1.0000. The log-binomial model almost always converged on the original data when the slope was zero. As the prevalence and slope increased, the percentage of times that the model converged declined.

The above analyses have involved a single quantitative variable, which allowed comparison to exact MLEs using the Deddens et al. macro, as well as giving an indication of when each method will be less biased than the other [12]. However, both methods will work for multiple independent variable models where the independent variables can be either categorical or quantitative. Our first example to illustrate this contains two quantitative independent variables (Table 3) [24]. The dependent variable is the vaso-constriction or non-constriction in digit skin. The independent variables are logarithms of rate and volume of inspired air. In this analysis, 20 of 39 observations (51 percent) were vaso-constricted. The p-values for the two methods are similar, and as expected, the standard errors of the log-binomial are smaller than those of the Robust Poisson. In addition, the estimates for the Robust Poisson are somewhat higher than those for the log-binomial, especially for the logarithm of volume. The estimated prevalence ratios for this variable were 2.16 for the log-binomial and 4.31 for the Robust Poisson. With the Robust Poisson, 3 of 39 estimated probabilities were greater than one, and the largest was 1.82.

**Table 3:** Comparison of log-binomial and Robust Poisson methods for analysis of vaso-constriction associated with inspired air*

Independent Variable	Log Prevalence Ratio Estimate^† ^(SE)		P-Value	
	
	Log-Binomial	Robust Poisson	Log-Binomial	Robust Poisson
Log(Rate)	1.3132 (0.3362)	1.5578 (0.4270)	0.0006	0.0003
Log(Volume)	0.7715 (0.1960)	1.4614 (0.3510)	0.0002	0.0000

* Wald tests were used for the Robust Poisson method, and likelihood ratio tests were used for the log-binomial method. The latter were obtained by fitting a model without the effect being tested. The log-binomial method failed to converge when both independent variables were in the model and when only log(Volume) was in the model. In these cases, the COPY method approximation was used.

^† ^The intercept estimate was -1.5147 for the log-binomial method and -1.8311 for the Robust Poisson method. Of the 39 probability estimates, 3 were greater than unity for the Robust Poisson method, and the largest was 1.82.

Our second example contains a three categorical variable, a two level categorical variable, and a quantitative variable (Table 4) [24]. The dependent variable is no pain. The independent variables are treatment (A, B, P), gender, and age. In this analysis, 35 of 60 patients (58%) had no pain. The p-values are somewhat different between the two methods, but the same conclusions would be drawn using either method. The estimate of the slope for age is nearly twice as steep for the Robust Poisson as for the log-binomial. The effect of gender is also doubled for the Robust Poisson, compared to the log-binomial. The effect of analgesic is similar for both methods. With the Robust Poisson, 9 of 60 estimated probabilities were greater than one, and the largest was 1.30.

**Table 4:** Comparison of log-binomial and Robust Poisson methods for analysis of no pain associated with covariates*

Independent Variable	Level	Log Prevalence Ratio Estimate^† ^(SE)		P-Value	
		
		Log-Binomial	Robust Poisson	Log-Binomial	Robust Poisson
Analgesic	A B	1.0228 (0.3951) 1.0979 (0.3898)	1.0628 (0.3902) 1.1515 (0.3882)	0.0002	0.0123
Gender	Female	0.2259 (0.0726)	0.4584 (0.1808)	0.0416	0.0112
Age		-0.0376 (0.0119)	-0.0635 (0.0183)	0.0075	0.0005

* Wald tests were used for the Robust Poisson method, and likelihood ratio tests were used for the log-binomial method. The latter were obtained by fitting a model without the effect being tested. The log-binomial method failed to converge for the 2 models containing both analgesic and age, and the COPY method approximation was used.

^† ^The intercept estimate was 1.1200 for the log-binomial method and 2.7438 for the Robust Poisson method. Of the 60 probability estimates, 9 were greater than unity for the Robust Poisson method, and the largest was 1.30.

Our third example contains 5 one degree of freedom independent variables (Table 5) [25,26]. The dependent variable is being given the death penalty. The independent variables are black defendant (yes, no), white victim (yes, no), seriousness of the crime, culpability, and culpability squared. In this analysis, 50 of 147 criminals (34%) were given the death penalty. This example will illustrate another issue with using the log-binomial model to estimate prevalence ratios instead of odds ratios. The quantitative variable culpability is linear in the log-odds but not in the log-probability. Thus one can use culpability in logistic regression, but in the log-binomial model we need to introduce a quadratic term. In general, if a variable is linear in logistic regression then it is not linear in the log-binomial model, and visa versa. Thus one should always test for linearity. In this example, the quadratic term in culpability is significant (assuming P(Type I error) = 0.05) in the log-binomial model (p = 0.0007) and for the Robust Poisson method (p = 0.0005), but it is not significant in logistic regression (p = 0.1246 with the likelihood ratio test). Of course this could also happen the other way (significant in logistic, but not in log-binomial). The estimates for the Robust Poisson are always larger in absolute value than for the log-binomial, and in some cases they are much larger. The same is true for the standard errors. With the Robust Poisson, 5 of 147 estimated probabilities were greater than one, and the largest was 1.28. Since the exponential model and the logistic model are fundamentally different, the notions of linearity, confounding, and interaction are not equivalent between logistic regression and log-binomial regression. For this reason, it is impossible to develop methods that convert adjusted odds ratios into adjusted prevalence ratios.

**Table 5:** Comparison of log-binomial and Robust Poisson methods for analysis of death penalty associated with covariates*

Independent Variable	Log Prevalence Ratio Estimate^† ^(SE)		P-Value	
	
	Log-Binomial	Robust Poisson	Log-Binomial	Robust Poisson
Black Defendant	0.3152(0.1367)	0.5935 (0.1992)	0.0224	0.0029
White Victim	0.1219 (0.1078)	0.3173 (0.2061)	0.2288	0.1238
Serious	-0.0010 (0.0174)	0.0023 (0.0352)	0.9305	0.9475
Culpability	1.8062 (0.2750)	1.9223 (0.4453)	0.0000	0.0000
Culpability Squared	-0.2006 (0.0308)	-0.2158 (0.0624)	0.0007	0.0005

* Wald tests were used for the Robust Poisson method, and likelihood ratio tests were used for the log-binomial method. The latter were obtained by fitting a model without the effect being tested. The log-binomial method failed to converge for all models containing Black Defendant. In these cases, the COPY method approximation was used.

^† ^The intercept estimate was -4.4445 for the log-binomial method and -4.9193 for the Robust Poisson method. Of the 147 probability estimates, 5 were greater than unity for the Robust Poisson method, and the largest was 1.28.

## Discussion

Maximum likelihood methods are very often the method of choice for estimating parameters because they are consistent, tend to have small variances, and are asymptotically unbiased and efficient. The log-binomial method evaluated in this paper obtains these MLEs, although when they are on the boundary of the parameter space, the estimates will be approximate. However if the number of copies is chosen large enough, the estimates will be the same as the true MLE rounded to several decimal places. Thus the log-binomial method should be expected to produce superior results when compared to the Robust Poisson if c is chosen large enough. The results in this paper show that c = 1000 is large enough to accomplish this.

The simulations included in the present paper involve only a quantitative independent variable. For qualitative variables, Skov et al. provided simulations and recommended the log-binomial method [11]. Skov et al. had no convergence problems with their qualitative data. Although convergence problems can occur with qualitative data, they are much more common with quantitative data, which makes it the more difficult and interesting case. The 3 real data examples in this study all contain at least 1 quantitative independent variable.

Our results from simulated data showed that both the Robust Poisson and the log-binomial method yielded estimates of the slope, and hence the prevalence ratio, which had little bias. For the common situations where the probability of success is between .3 and .7, the log-binomial method generally yielded less biased estimates and smaller standard errors than the Robust Poisson method. (For the somewhat unusual situation where the probability of success is .9, the Robust Poisson method was generally less biased.) Both the Robust Poisson method using the Wald test and the log-binomial method using the likelihood ratio test almost always had acceptable size, but the log-binomial method generally had higher power.

These simulations represent the typical performance of the two methods. In other simulations that we have done, we separated the 1000 replications into those for which the log-binomial model converged on the original data set and those for which it did not. Generally, when the log-binomial model converged, the estimates of the logs of the prevalence ratios were the same or close for the log-binomial and Robust Poisson methods to 3 decimal places. When the log-binomial model did not converge, however, the two methods were generally different to 3 decimal places. These simulations had a sample size of 100. For smaller sample sizes, the differences are larger. One such situation, which has already been published, is the following [12,23]. Suppose *X *takes on integer values between one and ten, inclusive, and that *Y *= 1 when *X *= 5, 7, 8, 9, or 10, and that *Y *= 0 otherwise. The exact MLE of the slope for the log binomial model is 0.2094. The log-binomial COPY method estimate is 0.2091, while the Robust Poisson estimate is 0.3251. In addition, the estimated P(*Y *= 1|*X *= 10) is 1.00 for the exact log-binomial MLE, 0.99 for the COPY method approximated MLE, and 1.44 for the Robust Poisson method. The latter is possible only because the wrong likelihood is being used.

For the real data, we do not know the correct parameters being estimated. However, the estimates are quite different for at least one variable in each of the 3 examples, and these differences will become larger when one takes the anti-log of the estimates to get estimated prevalence ratios. The Robust Poisson method again yields probability estimates which are greater than one. Because of these differences, the decision on which method to use should not be taken lightly. When it comes time to defend ones results, using the log-binomial model allows one to say that maximum likelihood estimation and likelihood ratio tests were used. Using the Robust Poisson, however, one must admit that the model is incorrect, and for some points, the predicted numerator of the prevalence ratio is not only incorrect, but invalid. One must also believe that the estimated denominator is incorrect so that the prevalence ratio can be correct.

Logistic analysis should not necessarily be ruled out even if one is interested in the prevalence ratio. Statistical tests may not be valid if too many terms are included in the model. The real examples given in this paper contain the maximum number of terms based on the commonly recommended rule of 10% of the number of events [28,29]. In the death penalty example, a quadratic term for culpability was required for the log-binomial model, but not for the logistic model. Thus both models yield estimated prevalence ratios which vary depending on the value of culpability. Thus one could reasonably argue that the more parsimonious logistic model might as well be used, if culpability is the variable of interest. In this example, most likely the variables of interest are defendant race and victim race, while culpability is a covariate for which the analysis should be adjusted. Thus the log-binomial model should probably be preferred for this example.

Spiegelman and Hertzmark recommend using the log-binomial when it converges but replacing it with the Robust Poisson when the log-binomial does not converge [30]. They illustrated the Robust Poisson part of the method on a set of real data for which the model converged. Deddens and Petersen responded that using the log-binomial when it converges but replacing it with the log-binomial on a data set modified by the COPY method when the original log-binomial did not converge (i.e. the log-binomial method used in this paper) was better in general, and showed that the COPY part of the method gave superior results to the Poisson part of their method on their data set [22].

We have shown by simulation that in most commonly occurring univariate cases, the maximum likelihood and approximate maximum likelihood estimates from the log-binomial method generally have an equal or smaller bias than do estimates from the Robust Poisson method. For the log-binomial model, we have only presented results for the likelihood ratio test. In general, the likelihood ratio test performs better than the Wald test, so using the correct model with the likelihood ratio test should be the best procedure. There is no reason to believe that the likelihood ratio test would be better for the Robust Poisson method because the method uses an incorrect likelihood. In fact, we have not found real or simulated data for which the Wald test performed poorly for the Robust Poisson method, but we have found (but not presented) such data for the log-binomial method.

When obtaining confidence intervals on the prevalence ratio, the Robust Poisson method will yield Wald based confidence intervals which will include 1.00 if and only if the two sided statistical test of H_0_:β_1 _= 0 is not rejected. At this time, if the model doesn't converge on the original data, and one uses physical copies, then SAS cannot be used to obtain likelihood ratio confidence intervals with the log-binomial method. However, Lumley et al. point out that physical copies are not necessary because one can do a weighted analysis [31]. Indeed, if one weights all observations in the original data set by (number of copies – 1)/(number of copies) and all observations in a modified data set by (1)/(number of copies), where the modified data set is simply the original data set with Y replaced by 1 – Y, then a weighted analysis of the combined data set yields results equivalent to those obtained with physical copies. With this weighted method, likelihood ratio confidence intervals for the log-binomial parameters can be obtained with SAS.

The Robust Poisson method solves the standard error problem of its non-robust predecessor [13,14]. In the rare case where the prevalence of interest is about 0.9, it may give less biased estimates than maximum likelihood methods. It is also easy to use for hypothesis testing and confidence intervals because it employs the Wald test. However, even if the form of the model is correct (log-binomial model), the estimated model may not be valid, which is easily seen when estimated probabilities for the points in the data set are greater than one. As shown by Skov et al., the log-binomial model produces estimates of probabilities which are between zero and one for any point in the convex hull of the covariates, which includes the observed data points [11]. Clearly the same is true for the COPY method modification because it uses the log-binomial method on the same covariates while changing from the exact solution, which is on the boundary of the parameter space, to one which is slightly to the inside of the boundary. We have used 1000 copies when the log-binomial model failed to converge. With more physical copies, simulations take a long time. Even when analyzing real data sets, using 10,000 physical copies may not be feasible if the original data set is very large. However, using Lumley et al.'s weighted modification, the number of copies is basically irrelevant because the data set size is only doubled for any weights [31]. As shown by Petersen and Deddens, 10,000 copies can yield estimates which are almost identical to the exact maximum likelihood estimates [22]. The Robust Poisson model can yield poor estimates in a few cases, and it generally has larger standard errors for the slope than the log-binomial method. Because likelihood ratio testing is possible for the log-binomial method (an advantage), multiple runs are required to get the p-values (a disadvantage). There are 2 things to note in this regard: 1) for large sample size, the Wald test worked well for the log-binomial method, and 2) if one doesn't need a p-value, one may be able to use the likelihood ratio confidence limits to determine statistical significance, which only requires one run. For programming languages other than SAS, the COPY method may still have convergence problems. Additional research with other software would be valuable.

## Conclusion

As shown with the real data used in this study, the results can be quite different depending on which method is used. Thus the decision on which method to use is very important. The simulations show that in the most common situations with a simple model, the maximum likelihood estimates of the log-binomial model are slightly superior to the Poisson based estimates. When the prevalence is very high, the Robust Poisson will have less bias than the log-binomial based methods, but it will yield many probability estimates greater than one. We believe that the advantages of the log-binomial method with the likelihood ratio test substantially outweigh those of the Robust Poisson when the true model is log-binomial. Future research could examine the effect of omission of terms and departures from the log-binomial model for both methods.

## Competing interests

The author(s) declare that they have no competing interests.

## Authors' contributions

MRP wrote the first draft of the manuscript, carried out the simulations and some other analysis, researched the topic, and prepared the manuscript for publication. JAD researched the topic, found the real data examples, ran some of the analyses, and gave detailed suggestions for revisions to the manuscript. Both authors read and approved the final manuscript. The authors performed this work as part of their official duties as employees of the National Institute for Occupational Safety and Health.

## Pre-publication history

The pre-publication history for this paper can be accessed here:


